# 2353: Competency-based training program for Research Professionals

**DOI:** 10.1017/cts.2017.174

**Published:** 2018-05-10

**Authors:** Megan Hoffman, Jennifer Maas, Lisa Johnson

**Affiliations:** University of Minnesota, Minneapolis, MN, USA

## Abstract

OBJECTIVES/SPECIFIC AIMS: To increase knowledge and application of clinical research coordinator competencies among Research Professionals at the University of Minnesota. METHODS/STUDY POPULATION: The UMN’s CTSI developed and piloted a Foundations for Research Professionals training program comprised of: a baseline assessment, 7 online modules, 4 in-person training sessions, video and reading assignments and a post assessment, which totaled 30–35 hours of training and covered the following topics: preparing for a study, study management, participant recruitment and engagement, assessing capacity to consent and the informed consent process. This course also provides valuable resources and connections to online references and materials. The competencies for this program were based on work of the Joint Task Force for Clinical Trial Competency. RESULTS/ANTICIPATED RESULTS: 30 clinical research professionals completed the pilot program and averaged an increase of 6.5% from baseline assessment to post assessment. Participants were asked to rate their confidence on a variety of role-based competencies at the time of preassessments and postassessments. Trends show an increase in confidence for all competency areas after completion of the training program. DISCUSSION/SIGNIFICANCE OF IMPACT: Developing a workforce of competent research professionals is integral to improve the efficiency, quality, and ethics of research. The Foundations for Research Professionals training program increased knowledge of clinical research coordinator competencies. We will assess impact on application of the competencies 6 months after completion of the program. Our next steps include offering the training program as a 2-week session on an ongoing basis for new coordinators at the University of Minnesota.

